# Report of the Physician to the Washingtonian Home of Chicago for 1867

**Published:** 1868-02

**Authors:** T. D. Fitch

**Affiliations:** Physician


ARTICLE VI.

### REPORT OF THE PHYSICIAN TO THE WASHINGTONIAN HOME OF CHICAGO FOR 1867.

*To the Executive Committee of the Washingtonian Home,*

GENTLEMEN:—I hereby beg leave to submit my Annual Report, as Physician of the Home, for the year ending December 31st, 1867:

There have been 45 cases of delirium tremens treated during the year, out of which only 21 have required active medical treatment. Many others have had no delirium tremens, but have suffered from various other morbid conditions consequent upon the use of alcoholic stimulants, such as paralysis, dropsy (general and local), uræmia, and one case of mental disorder amounting to insanity. This case was discharged as incurable, and is one of uncommon interest.

There has been but one death during the year, that of Joseph Cunningham, who has an interesting history, which will be related by the Superintendent. He was admitted on the 27th day of June, in the active stage of delirium tremens, and died on the 30th, from uræmic convulsions. There was suppression of urine from the day of his admission, and partial suppression for some time previous. His eyes were congested; the entire surface of the body presented a bloated appearance, amounting to anasarca or general dropsy of the cellular tissue; he was raving and wakeful, and all the means I could employ failed to procure sleep or rest; he could not, or would not, take nourishment, and died a most horrible death in convulsions.

The next day after, a *post mortem* examination was made by Dr. H. M. Lyman and myself, in the presence of a large number of the inmates of the Home, who were permitted to witness the examination, that they might, with their own eyes, see the destructive effects of alcohol on the human system, which, in our opinion, although silent, was a most impressive *lecture.*

The heart was found to be large, its walls thin and soft; an unusual amount of serum was found in the pericardium and pleura; the lungs were engorged with blood; the liver was found enlarged, nodulated, and friable, being easily broken down or torn, presenting the appearance of what is called a "*whiskey liver;*" the stomach was partially filled with a dark grumous fluid; but the most interesting feature of the case was, that the mucous membrane was highly inflamed and softened, presenting the characteristic appearances of a drunkard's stomach, as given by Dr. Sewell, in his plates showing the condition of this membrane, from the moderate drinker to the confirmed drunkard. This stomach corresponded most perfectly with that of the confirmed drunkard. The stomach was presented to Dr. N. S. Davis, a member of your Committee. The kidneys were found changed in their structure by what is called fatty degeneration, so that they were unable to perform their natural functions, hence, the retention in the blood of the poisonous elements of the urine, which acted as a direct poison on the brain, resulting in convulsions and death.

We regarded his case as incurable at the time of his admission. He had good care and close attention. A number of similar cases have been treated at the Home during the year, but have recovered, though none had progressed so far as his.

Another case is worthy of special mention, that of J. F., who had no delirium tremens, but suffered from general dropsy, caused from an impoverished condition of the blood. A large amount of serum was effused into the pleural and pericardial cavities, and a general anasarcal condition of the cellular tissue. He was kept quiet; tonics, iron, and diuretics were given, together with a nourishing diet, and his recovery was rapid and permanent.

The general treatment in cases of delirium tremens has been mainly the same as last year. Patients, on admission, have been bathed and kept clean. If constipation existed, as it generally does, a mild cathartic or laxative was administered. The urinary secretions were carefully watched, believing, as we do, that much of the nervous disturbance is caused by retention in the blood of the poisonous elements of the urine. To relieve restlessness and sleeplessness, we have found the bromide of potassium, in doses varying from 10 to 40 grains every three hours, the most generally applicable and effectual. One patient, who had had many sieges of delirium tremens, was so promptly relieved by this medicine, that he exclaimed, "that is the best medicine in the world, there was never such another." Chloroform, opium, opium and quinia, lupulin, digitalis, etc., etc., were occasionally used, and other remedies, as complications, which will always arise, seemed to indicate.

No alcoholic or kindred stimulants have been allowed at the Home. All stimulants have been withdrawn at once, and you will see, by the small amount of deaths compared with the whole number treated, gives a very flattering success. Notwithstanding there are many who recommend the "tapering off plan," I am prepared to assert my convictions that the "sudden jerk plan," as termed by the *Cincinnati Daily Commercial*, is the most successful, and should be adopted by all institutions designed for the reformation of inebriates.

I am happy to report that the sanitary condition of the Home has been much improved over the preceding year. Good sewerage, which was deficient last year, is now complete. Better accommodations have been provided for the sick, and better and more faithful care and attention given the patients. The Hospital Department has been materially enlarged, and better ventilation secured.

The matter of furnishing employment for the convalescents and fully recovered inmates, as suggested in my last annual report, and as provided for in our new constitution (or charter), has been referred to a committee, with power to act in the adoption of such employments as shall at once be profitable for the Home and conducive to the health of the inmates. All of which is respectfully submitted.

T. D. FITCH, M.D., *Physician.*

